# The Life Problem at Forty

**Published:** 1890-11-01

**Authors:** 


### The Doctor's Armchair.

### THE LIFE PROBLEM AT FORTY.

The man who is born rich, or high in rank, or without ambition, seldom understands or has such a keen relish of life as he who is the architect of his own fortunes. The spectators at Lord's enjoy the game in a languid sort of way, and are sometimes roused to the enthusiasm of a tepid "cheer"; but their pleasure is as nothing compared with the strenuous delight of a Grace, or the victorious determination of a Lohmann or a Spofforth. To know what a battle is the soldier must fight; and to understand the pleasures and pains of love a man must have a lover and a rival. Many a school boy, wearied of his books and of the restraints of authority, looks eagerly forward to the dawn of the day of freedom and of the practical struggle for life and advancement. At twenty the human race-horse can hardly be reined in till the moment for starting arrives; at forty the marks of hard galloping and of a great variety of stable experiences are everywhere visible. But at forty no man of spirit and sense, whose chances have been moderately fair, can be compared with a worn-out race-horse. If we may continue to use metaphor we would say that he should be a weight-carrying hunter, in the very prime of his age, whose work in life has taken little from his speed, whilst it has brought his fencing "cleverness," and his staying power to the highest possible degree of perfection. If a man be not a fool he ought to have so trained himself that the period between forty and sixty-five or seventy, shall produce the largest quantity and the highest quality of work which he has it in him to put forth.

Forty is a critical age with most men. Some have run their last competitive race by that time, and are doomed for the rest of their lives to a sober trot, which shall gradually degenerate into a spiritless and melancholy walk. Either from a want of inherent vitality and high courage, or from the too oppressive force of circumstances, their most strenuous efforts have all been made; and have all failed to separate them from the struggling crowd whose names and features are doomed to final oblivion. But all men are not extinguished at forty; some are never extinguished; and no man who reads the secret of life aright, let his circumstances be as obscure and oppressive as they may, ever permits himself to be finally extinguished at all. An intellectual being like man owes it to his Creator and to himself to preserve in his soul an immortal fountain of new purpose, new effort, and new hope. The man who is incapable of a new purpose and a new hope is already intellectually dead.

In politics, in religion, in medicine, and in all the activities that demand brain power and moral energy, the best and most critical work falls upon the shoulders of men of mature mind and years. Very few men are intellectually mature before the age of forty. Indeed, the highest point of physical health is not always touched before that period. It is true that many have exhausted and worn-out their bodies, as others have dissipated the energies of their minds, long before that time. But those have been mostly fools, and must reap the fool's reward. The men of worthier intellectual and physical stuff have preserved both body and mind in health; and have made the first twenty years of their manhood a period of training, not of all-round squandering and waste. Does any reader of forty or forty-five find his mental and bodily finances in an embarassed and partially exhausted condition? If he does he may assure himself that he has been either a very unwise or a very unlucky man.

The best half of life is in front of the man of forty, if he be anything of a man. The work he will do will be done with the hand of a master, and not of a raw apprentice. The trained intellect does not see "men as trees walking," but sees everything clearly and in just measure. The trained temper does not rush at work like a blind bull at a haystack; but advances with the calm and ordered pace of conscious power and deliberate determination. To no man is the world so new, and the future so fresh, as to him who has spent the early years of his manhood in striving to understand the deeper problems of science and life, and who has made some headway towards comprehending them. To him the commonest things are rare and wonderful, both in themselves, and as parts of a beautiful and intelligent whole. Such a thing as staleness in life and its duties he cannot understand. Knowledge is always opening out before him in wider expanses, and more commanding heights. If he be a doctor he finds, for example, that the fresh study of one of the organs of the human body yields a pleasure and an enlightenment which it never yielded before; he sees the wonders of minute structure with new powers of comparison; he marks the relations that subsist between different organs with fresh comprehension and delight; he sees possibilities of development, and of restoration, which never struck him in his earlier studies; the various parts of the body, and the whole of the organism in relation to its powers, its functions and its environment, constitute a miracle, the wonderfulness of which knows no end. The pleasure of growing knowledge and increasing power in his peculiar field of work, makes every year of his life happier and more hopeful than the last.

Some men of action, who know little of science and less of literature, begin to find the world and their daily occupations very stale after the age of forty. If they are making money pretty quickly, they account that something of a compensation; but if they merely earn enough to enable them to pay the butcher's bills and the rent when it becomes due, and have no hope of reaching a stage of greater enlargement and prosperity by-and-bye, they are very apt to yield to the temptation of a slowly increasing hopelessness. Such men experience a throb of sympathy when they hear a literary man or preacher ask the now familiar and mean-spirited question: "Is life worth living?" To them the true secret of life is still unrevealed. Eternal youthfulness is the thing to be sought after, and it is quite possible to be gained. The man who has found out the secret of it, and who keeps firmly hold of his talisman, is assured of a progressively growing and strenthening intellect, and an increasing victory and success in life. The success may not always be that which he originally set out to achieve. But, such as it is, when he has gained it, he himself will be the first to affirm that it is not only as good but better than what he wished for in the days of his inexperience. In a word, to the man who has comprehended life aright, the age of thirty is better than that of twenty, forty is better than thirty, and fifty better than forty. The old man who has reaped the fullest and best harvest that providence has sown for intelligent and moral creatures is more youthful, more hopeful, and more joyful than the most vigorous and exuberant youth, who has failed to select the richest crops that lie ready to fall to his scythe. This is not a conundrum, or a flight of enthusiasm, but a sober result of careful scientific observation and comparison.

